# Establishment of the reproducible branch retinal artery occlusion mouse model and intravital longitudinal imaging of the retinal CX3CR1-GFP^+^ cells after spontaneous arterial recanalization

**DOI:** 10.3389/fmed.2022.897800

**Published:** 2022-07-15

**Authors:** Jehwi Jeon, Sang-Hoon Kim, Eunji Kong, Soo Jin Kim, Jee Myung Yang, Joo Yong Lee, Junyeop Lee, You-Me Kim, Pilhan Kim

**Affiliations:** ^1^Graduate School of Medical Science and Engineering, Korea Advanced Institute of Science and Technology (KAIST), Daejeon, South Korea; ^2^KI for Health Science and Technology (KIHST), Korea Advanced Institute of Science and Technology (KAIST), Daejeon, South Korea; ^3^Asan Medical Center, University of Ulsan College of Medicine, Seoul, South Korea; ^4^Dongguk University Ilsan Hospital, Ilsan, South Korea

**Keywords:** branch retinal artery occlusion (BRAO), mouse modeling, ischemic-reperfusion injury, retinal microglia, intravital imaging

## Abstract

Animal models of retinal artery occlusion (RAO) have been widely used in many studies. However, most of these studies prefer using a central retinal artery occlusion (CRAO) which is a typical global ischemia model of the retina, due to the technical limitation of producing single vessel targeted modeling with real-time imaging. A focal ischemia model, such as branch retinal artery occlusion (BRAO), is also needed for explaining interactions, including the immunological reaction between the ischemic retina and adjacent healthy retina. Accordingly, a relevant model for clinical RAO patients has been demanded to understand the pathophysiology of the RAO disease. Herein, we establish a convenient BRAO mouse model to research the focal reaction of the retina. As a photo-thrombotic agent, Rose bengal was intravenously injected into 7 week-old transgenic mice (CX3CR1-GFP) for making embolism occlusion, which causes pathology similarly to clinical cases. In an optimized condition, a 561 nm laser (13.1 mw) was projected to a targeted vessel to induce photo-thrombosis for 27 s by custom-built retinal confocal microscopy. Compared to previous BRAO models, the procedures of thrombosis generation were naturally and minimal invasively generated with real-time retinal imaging. In addition, by utilizing the self-remission characteristics of Rose bengal thrombus, a reflow of the BRAO with immunological reactions of the CX3CR1-GFP^+^ inflammatory cells such as the retinal microglia and monocytes was monitored and analyzed. In this models, reperfusion began on day 3 after modeling. Simultaneously, the activation of CX3CR1-GFP^+^ inflammatory cells, including the increase of activation marker and morphologic change, was confirmed by immunohistochemical (IHC) staining and quantitative real-time PCR. CD86 and Nox2 were prominently expressed on day 3 after the modeling. At day 7, blood flow was almost restored in the large vessels. CX3CR1-GFP^+^ populations in both superficial and deep layers of the retina also increased around even in the BRAO peri-ischemic area. In summary, this study successfully establishes a reproducible BRAO modeling method with convenient capabilities of easily controllable time points and selection of a specific single vessel. It can be a useful tool to analyze the behavior of inflammatory cell after spontaneous arterial recanalization in BRAO and further investigate the pathophysiology of BRAO.

## Introduction

There have been many studies simulating an ischemia by the retinal artery occlusion (RAO) modeling (1). Typically, ischemia models are divided into global and focal ischemia (2–4). However, most previous studies focus on global ischemia (5). For example, increase the intra-ocular pressure (6–8), ligation of the ophthalmic artery (9, 10) and administration of the vasoconstrictor (11, 12) were used for global ischemia models of central retinal artery occlusion (CRAO). However, a pathologic mechanism of an extreme increase of the intraocular pressure more resembles that of acute glaucoma than CRAO. Ligation of the ophthalmic artery model cannot remove the effect of choroidal ischemia, such as ocular ischemic syndrome. Lastly, administration of the vasoconstrictor is a more preferred model for amaurosis fugax, transient vision loss (13). Yet, despite relatively low clinical relevance and limitations, these global ischemia models have been mainly used to study RAO due to a technical convenience.

In this study, we focused on a focal ischemia model, similar but prominently different from the global ischemia. The focal ischemia models can not only provide information of the occluded vessel size or ischemic area size, but also provide an interaction between the ischemic retina and adjacent healthy retina. This is quite useful for the analysis of immunologic processes, such as the glial reaction of the ischemia in the retina. Currently, an argon photo-coagulation model (14, 15) has been widely used for a focal ischemia model in the ophthalmologic area. However, this can produce permanent vessel occlusion as an inevitable consequence of large photo-mechanical damage, thereby posing difficulty in analysis of ischemic damage. On the other hand, photo-thrombosis induced stroke model using the Rose bengal as a photo-thrombotic agent has been well established (16–19). Under laser illumination, the intravenously injected Rose bengal produces reactive oxygen species such as singlex oxygen radicals, which activates tissue factor and subsequently initiates coagulation cascade: platelet aggregation, thrombus formation and vessel occlusion. Additionally, while a real-time animal single vessel stroke model by photo-thrombosis is already well known (20), single vessel stroke animal modeling has been technically challenging in the retina.

The focal ischemia of retina is a branch retinal artery occlusion (BRAO). Without treatments, serious outcomes are expected with this ischemia. However, there are very limited therapeutic options to care for the diseases well. Direct interventional therapies have been chosen as a treatment to avoid serious outcomes of this disease in clinics. Tissue-type plasminogen activator (tPA) thrombolytic therapy has been managed for RAO patients like the percutaneous coronary intervention (21). Unfortunately, the reperfusion by the thrombolytic therapy causes additional damage to cells, significantly more than ischemia alone. Thus, much research about this ischemia-reperfusion (IR) injury have been conducted in multiple fields, including cardiology and neurology (22, 23). There are also several reports that microglia is activated by IR injury in cerebral infarction (24–26). However, the dynamics of retinal microglia mediating IR injury has not been well investigated yet.

Until now, the RAO studies using various models have not showed the natural embolism infarction of the retinal artery in real-time imaging (11, 27–29). Herein, to reintroduce natural BRAO in clinics, we implemented a more natural and improved BRAO modeling by using our previously established custom-built high-speed multicolor confocal microscopy and Rose bengal as a photo-thrombotic agent. We show the real-time imaging of repetitive and single vessel specific thrombus making for the first time. This study also presents serial imaging of BRAO models to confirm this pathophysiology for 7 days. We focused on day 3 and 7 for analyzing CX3CR1-GFP^+^ inflammatory cell changes after the time point of the reperfusion.

## Materials and methods

### Custom-built high-speed laser-scanning retinal confocal microscopy

**FIGURE 1:**
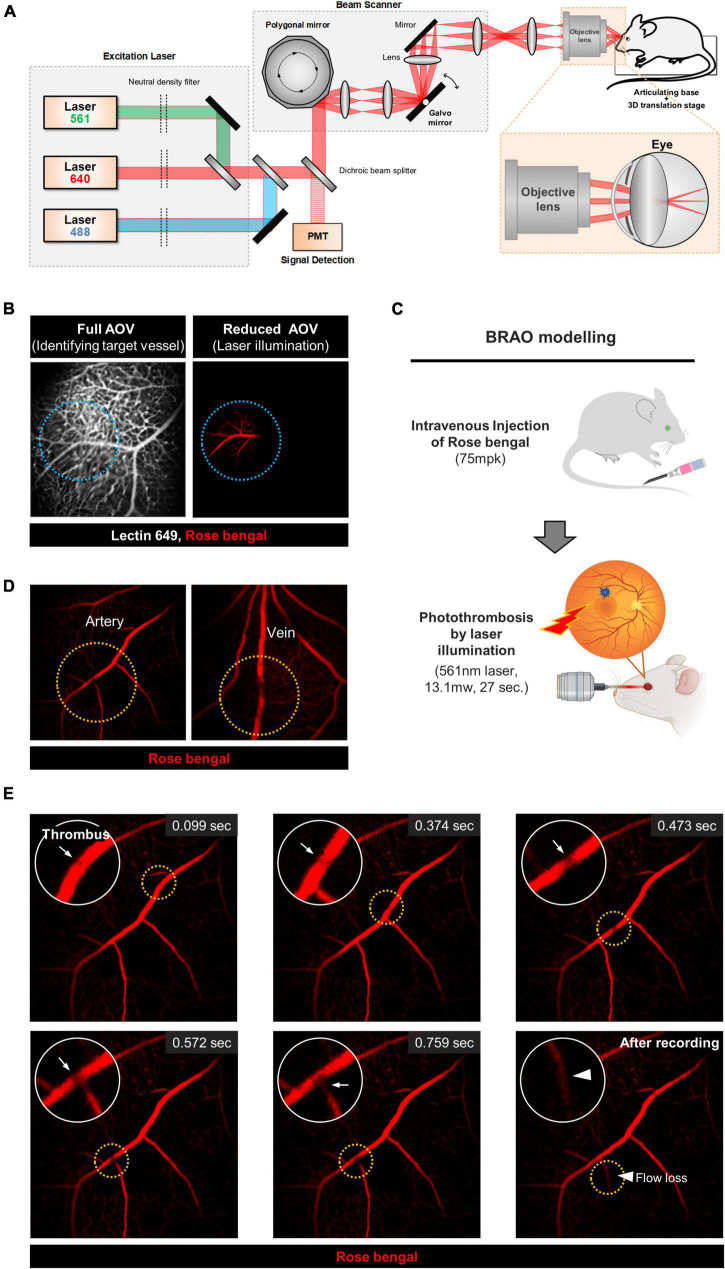
The schematic of custom built confocal microscope and the real-time BRAO modeling. **(A)** A schematic of the custom-built, high-speed, laser-scanning confocal microscope for intravital retinal imaging. **(B)** Selection of the artery for BRAO modeling. Actual illuminated area is outlined by *the blue dotted line*. **(C)** A schematic of the brief method for BRAO modeling. **(D)** Thrombosis can be made in both large artery and vein. Thrombus sites are high-lighted by *orange dotted line*. **(E)** Representative real-time images of the BRAO modeling. *Arrows* indicated thrombus. *Arrow head* indicated blood flow loss. BRAO, branch retinal artery occlusion.

A previously described custom-built high-speed laser-scanning retinal confocal microscopy was used (30–32). The schematic of the system is depicted in Figure 1A. Three continuous-wave laser sources, composed of a 488-nm diode laser module (MLD488; Cobolt AB, Stockholm, Sweden), 561-nm DPSS laser (Jive; Cobolt), and 640-nm diode laser module (MLD640; Cobolt) were used as excitation light sources. Laser beams from laser modules were expanded by telescopes consisting of an achromatic lens with focal lengths of 45 and 125 mm for a 561-nm wavelength laser beam (#47-636, #47-642; Edmund Optics, Barrington, NJ, United States) or 45 and 150 mm for 488-nm and 640-nm wavelength laser beams (#47-636, #47-643; Edmund Optics). Intensity of each laser was independently adjusted by a continuously variable neutral density filter (NDC-50C-4M-A; Thorlabs Inc., Newton, NJ, United States). All three laser beams were combined by using dichroic beam splitters (FF593-Di03, FF520-Di02; Semrock Inc., Rochester, NY, United States) and then delivered to a beam scanner by a multiedge dichroic beam splitter (Di01-R405/488/561/635; Semrock). Two-dimensional Raster scanning at 90 Hz was achieved by the beam scanner composed of a rotating 72-facet enhanced aluminum-coated polygonal mirror (SA24; Lincoln Laser, Phoenix, AZ, United States) for fast-axis scanning and a galvanometer mirror scanner (6230H; Cambridge Technology, Bedford, MA, United States) for slow-axis scanning. To implement telecentric scanning system, achromatic lenses with effective focal lengths of 50 mm (#47-637; Edmund Optics), 75 mm (#47-639; Edmund Optics), 75 mm with an aperture of 2 inches (#49-292; Edmund Optics), and 125 mm (#47-642; Edmund Optics) were used. Finally, the scanning laser beams were focused by the crystalline lens in the eye of an anesthetized mouse and delivered to the retina through a commercial objective lens (PlanApoλ20X, 0.75NA; Nikon Corporation, Tokyo, Japan). The anesthetized mouse was placed on the articulating-baseball stage (SL20; Thorlabs) fixed to the XYZ translation stage (3DMS; Sutter Instrument, Novato, CA, United States). Fluorescence signals were collected by the objective lens. Descanned fluorescence signals were separated from the excitation laser beams by one dichroic beam splitter and split into three individual fluorescence signals (green, red, and far-red) by the other dichroic beam splitters (FF560-Di01, FF649-Di01; Semrock). Each fluorescence signal was detected by a photomultiplier tube (PMT; R9110; Hamamatsu, Shizuoka Prefecture, Japan) through bandpass filters (FF01-525/45, FF01- 600/37, FF01-697/58; Semrock), 75-mm focal-length achromatic lens (#47-639; Edmund Optics), and confocal pinholes. Electronic signals from the PMTs were simultaneously digitized by using three-channel frame grabber (Solios; Matrox, QC, Canada) with a sampling rate of 29.86 MHz for each channel. Finally, multicolor real-time images with a frame size of 512 by 512 pixels were displayed and recorded at the frame rate of 90 Hz by a custom-developed imaging software using Matrox Imaging Library (MIL9; Matrox) (33, 34).

### Animal models

All animal experiments were approved by the Institutional Animal Care and Use Committee of Korea Advanced Institute of Science and Technology (KAIST) (approval No. KA2021-003). All animals were treated, maintained, and sacrificed in accordance with the policies specified in the ARVO Statement for the Use of Animals in Ophthalmic and Vision Research. Mice were housed and bred in an institutional animal facility in KAIST. All mice were individually housed in ventilated and temperature- and humidity-controlled cages (22.5°C, 52.5%) under a 12/12-h light/dark cycle and provided with standard diet and water *ad libitum*. For experimental use, C57B6/N mice were purchased from OrientBio (Suwon, Korea). CX3CR1-GFP, Thy1-YFP-16 mice were purchased from Jackson Laboratory (Stock No: 005582 and 003709, Bar harbor, ME, United States). To induce BRAO, 75 mg/kg dose of the Rose bengal (stock no: 330000, Sigma-Aldrich, Saint Louis, MO, United States) was delivered by tail vein injection (35). Then the 561-nm DPSS laser (Jive; Cobolt) beam was projected to the target vessels for 27 s with 13.1 mw power. To induce RPE cells degeneration for outer blood-retina barrier (BRB) break, sodium iodate (NaIO_3_, 50 mg/kg, stock no: S4007, Sigma-Aldrich) was injection *via* peritoneum in 0.05% acetic acid solution (Stock no: A6283, Sigma-Aldrich) (36, 37).

### Intravital retinal imaging of mouse retina

For intravital imaging, the mouse was also anesthetized with a mixture of zoletil (30 mg/kg) and xylazine (10 mg/kg) by intramuscular injection. Body temperature of the anesthetized mouse was maintained at 36°C by using a homeothermic temperature monitoring and control system (RightTemp; Kent Scientific, Torrington, CT, United States) to prevent the abrupt formation of cold cataract hampering the imaging of retina. Yohimbine (2 mg/kg), antagonist of xylazine, was injected to provide protection from corneal injury or dryness during post-anesthesia recovery and stabilization of cardiovascular systems. Additional protective measures, including eye ointment and artificial tear to avoid corneal injury, were used with an infrared heating lamp during the recovery state from anesthesia. To visualize retinal vasculature, 25 μg anti-CD31 antibody (stock no: 553708, BD Biosciences, Franklin Lakes, NJ, United States) conjugated with a far-red color fluorophore, Alexa Fluor 647 (stock no: A20006, Invitrogen, Waltham, MA, United States) or lectin DyLight 649 (DL-1178, Vector laboratories, Burlingame, CA, United States) were intravenously injected, which fluorescently labeled endothelial cells of the whole body in a systemic manner. To visualize current flows of the retinal vasculature, TRITC-dextran 100 mg/kg (tetramethylrhodamine isothiocyanate dextran 155 k, T1287, Sigma-Aldrich) was simultaneously intravenously injected with endothelial labeling.

### Histology procedure for immunohistochemistry of retinal tissues

After the intravital retinal imaging, mice were euthanized by using a CO_2_ chamber. Both whole eyeballs with optic nerves were carefully harvested by using forceps without tearing and immersed in 1% paraformaldehyde solution (16%, 30525-89-4, MP Biomedicals, CA, United States, diluted in PBS) during over-night for the fixation of entire tissue. After washing the fixed eyeballs with PBS, they were placed in a small cell culture dish under stereoscopic microscope. Linear incision was made by No. 11 blade at the center of the cornea. Subsequently, a circular incision was performed through the limbus by using iris scissors and the cornea was detached. Crystalline lens, iris, choroid, and sclera were stripped off and the optic nerve was gently cut while avoiding tangential traction damage in the retina. Vitreous body and firmly attached ciliary body were carefully and totally removed from the retina. And neuro-retinal fraction and choroid-RPE fraction were divided through subretinal space dissection. Each tissues was trimmed to be made four-leaf clover shape for visualizing *en face* flat-mounted image.

The processed retinas (flat-mounted and sliced) were blocked in 5% normal goat serum in PBST (0.3% Triton X-100 in PBS) and incubated overnight at 4°C with the following primary antibodies (1:200): anti-CD31 (PECAM, rat, stock no: 553708, BD Biosciences), anti-CD86 (rabbit, stock no: ab242142, Abcam Inc., Cambridge, United Kingdom), anti-Iba1 (rabbit, stock no: Cat #PA5-27436, Thermo Fisher Scientific, Waltham, MA, United States) and anti-CCR2 (rabbit, stock no: ab245898, Abcam). After washing 8 times in 0.3% PBST, the samples were incubated for 2 h incubation at RT in shaker with species-specific secondary Alexa Fluor-coupled secondary antibodies (1:250) in 0.3% PBST solution (Goat anti rat A555 secondary antibody: A21434, Thermo Fisher Scientific, Goat anti rat A647 secondary antibody: ab150167, Abcam, Goat anti rabbit A555: A32732, Goat anti rabbit A647: A32733, Thermo Fisher Scientific). Subsequently, the samples were washed in 4 times 0.3% PBST and then 4 times PBS, respectively, and placed in a slide glass with mounting medium (VECTASHIELD^®^ Anti-fade Mounting Medium, H-1000-10, Vector laboratories). Immunofluorescence images were acquired using a custom-built confocal microscopy as described in the section “Custom-Built High-Speed Laser-Scanning Retinal Confocal Microscopy.”

### RNA isolation and quantitative real-time PCR

Eyes were collected from anesthetized mouse and quickly dissected by a circumferential incision through limbus in cold RPMI to alleviate cell viability. The neuro-retina was carefully harvested from choroid and sclera. And total RNA from the neuro-retina fraction were extracted and purified using the RNA-spinTM (Intron biotechnology, Seongnam, Gyeonggi-do, South Korea) following the manufacturer’s protocol. The quantity and quality of total RNA from each sample was analyzed using the Agilent Bio-Analyzer and Agilent RNA 6000 pico kit (stock no: #5067-1513, Agilent, Santa Clara, CA, United States). The reverse transcription of mRNA was performed by using the GoScript™ Reverse Transcriptase kit (stock no: A5001, Promega, Madison, WI, United States) to make cDNA library. Reactions were performed in 30 μl volumes containing 5× GoScript ™ 5X Reaction Buffer, 25 mM of MgCl2, 10 nM of PCR nucleotide mix, 500 μg/ml of Oligo primer, GoScript™ Reverse Transcriptase, 40 u/μl of Recombinant RNasin^®^ Ribonuclease Inhibitor, Nuclease-Free Water and 1.5 μl of the mRNA product. RT-PCR were carried out as follows: 5 min at 25°C (annealing), 60 min at 42°C (extension), and 15 min at 72°C (inactivation RT). The following SYBR green assays (QPK-201, Toyobo, Osaka, Honshu, Japan) were performed for RT-PCR by Bio-rad qPCR machine (Bio-rad, Hercules, CA, United States) by using the prepped cDNA library. And there are primer list used in the experiment at Supplementary Table 1. This reaction was performed in 20 μl volumes containing 8 pM of each primer mentioned above, 6.4 μl of RNase-Free Water, 10 μl of the SYBR green and 1.0 μl of the cDNA product. Quantitative real-time PCR were carried out as follows: 15 s at 95°C (denaturation), 15 s at 60°C (annealing), and 30 s at 72°C (extension), these steps were repeated for 45 cycles.

### Image quantification analysis and statistical analysis

The calculation of reperfusion/ischemic area ratio in vascular reperfusion was done by utilizing “surface” function of commercial image analysis software, IMARIS (ver 9.02, Bitplane, Belfast, United Kingdom). The ischemic area of the day 1 was selected as a reference region of the interest (ROI) for day 3 and 7. Then, “surface detail” value of the area was determined as 1 μm, and the minimum and maximum cut-off values were set to 20.0 and 30.0, respectively. The number of inflammatory cells was also calculated with same IMARIS software. The inflammatory cells with above 12.5 μm of the estimated X-Y diameter in “spot” function was considered. And a filter type was selected as “quality” (lower threshold: 4.0, upper threshold: 15.0 in ROI). CX3CR1-GFP^+^ cells were regarded as activated when they showed one of the following features: retraction of their processes lost their “tile span” distribution, increased population, and featured amoeboid shape. To quantify activated CX3CR1-GFP^+^ cells, individual inflammatory cells were automatically identified as those with a soma size of above 22.5 μm by using the “Spot” function in the IMARIS. An activated “amoeboid shape” microglia was defined as that with a soma size of more than 50 μm and dendrite size below 50 μm. Statistical analysis was performed by using IBM SPSS Statistics 26.0 (SPSS, Inc., Chicago, IL, United States). Statistical difference was determined by a non-parametric Mann Whitney *u*-test. The R square was calculated by simple regression analysis. Multiple comparison among groups were determined by one-way ANOVA followed by Dunnett’s *post-hoc* test. A statistical significance was set at *P*-values less than 0.05.

## Results

### Establishment of a reproducible branch retinal artery occlusion modeling method

Real-time intravital images and movies of the BRAO model were acquired by using a previously established confocal microscopy setup (30, 34, 38), as shown in Figure 1A. First, a target vessel labeled by lectin 649 staining was selected in the maximum angle of view (AOV), which was previously measured to be about 48°. The AOV was then reduced to be a suitable size for a single branch occlusion of the retinal artery by photothrombosis with 561 nm laser illumination, as shown in Figure 1B. This adjustment of AOV was controlled by changing the distance between the objective lens and mouse crystalline lens in the range of 10 ∼ 15 mm. The used AOV for BRAO modeling was 16.4∼22.4°, depending on target vessel size. With repeated trial of the modeling with different parameter setting, the 561 nm laser projection parameters to induce photo-thrombus in a single targeted vessel with expected reperfusion at day 3 were empirically determined to 27 s with 13.1 mW power (Figure 1C). With this optimized laser power and duration, the Rose bengal thrombus is effectively formed and maintained in a single retinal vessel for a relatively short period of time unlike other photo-thrombosis models (39, 40). Additionally, no subretinal hemorrhages and vessel ruptures were observed after the laser illumination. Notably, the target vessel for occlusion can be readily selected between artery and vein, as depicted in Figure 1D, which can be easily identified by flowing direction and vessel morphology. A real-time intravital imaging clearly showed the whole processes of the BRAO modeling from thrombosis to blood flow loss of the occluded vessel, as shown in Figure 1E and Supplementary Movie 1. Briefly, we successfully established a standardized and reproducible BRAO modeling method.

### Serial longitudinal intravital imaging of the branch retinal artery occlusion models

**FIGURE 2:**
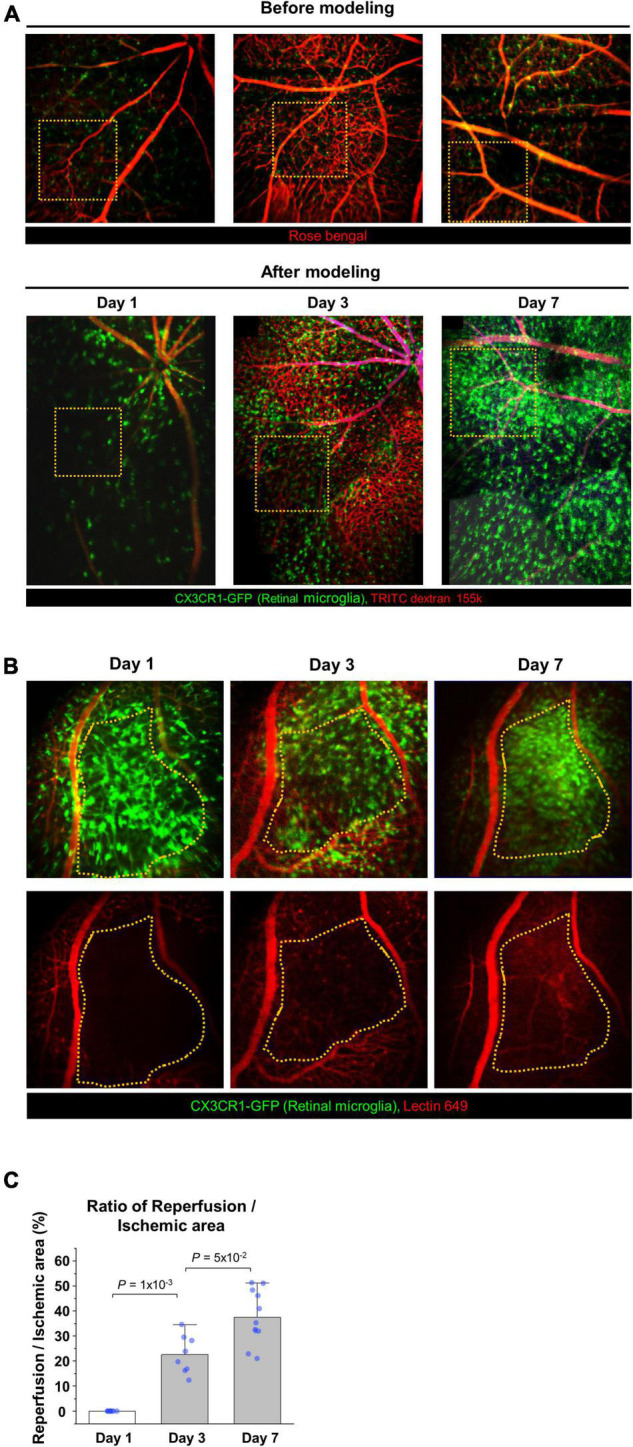
Serial intravital imaging of the blood flow in BRAO modeling. **(A)** Overall changes of the large vessels blood flow and CX3CR1 signal expression for 7 days. The images used in before modeling were single AOV image. The images used in after modeling were stitched intravital mosaic images. Target arteries were outlined by *orange dotted box*. **(B)** Serial intravital images of the vessel reperfusion in the same mouse. Targeted ischemic area were outlined by *orange dotted line*. **(C)** Quantification of the reperfusion/Ischemic area (%) in **(B)**. (*n* = above 8, retinas per each group). AOV, angle of view.

To analyze the changes of blood flow and immunologic reaction in the established ischemic BRAO model, intravital images and histology images of the occluded vessels and inflammatory cell changes were serially acquired by using CX3CR1-GFP mouse (41). These serially acquired intravital mosaic images were obtained at the modeling day and then day 1, 3, 7 post modeling, respectively, as shown in Figure 2A. Right after BRAO modeling, the occluded area could be easily identified as a non-perfusion area at day 1. Re-flow started to be observed in the occluded ischemic area at day 3, and then the retinal blood flow was gradually recovered. Finally, the retinal blood flow of the large blood vessel in the occluded area was almost entirely recovered at day 7. Figures 2B,C show the progress of the reperfusion around the BRAO ischemic site and the quantification of the ratio of reperfusion/ischemic area.

**FIGURE 3:**
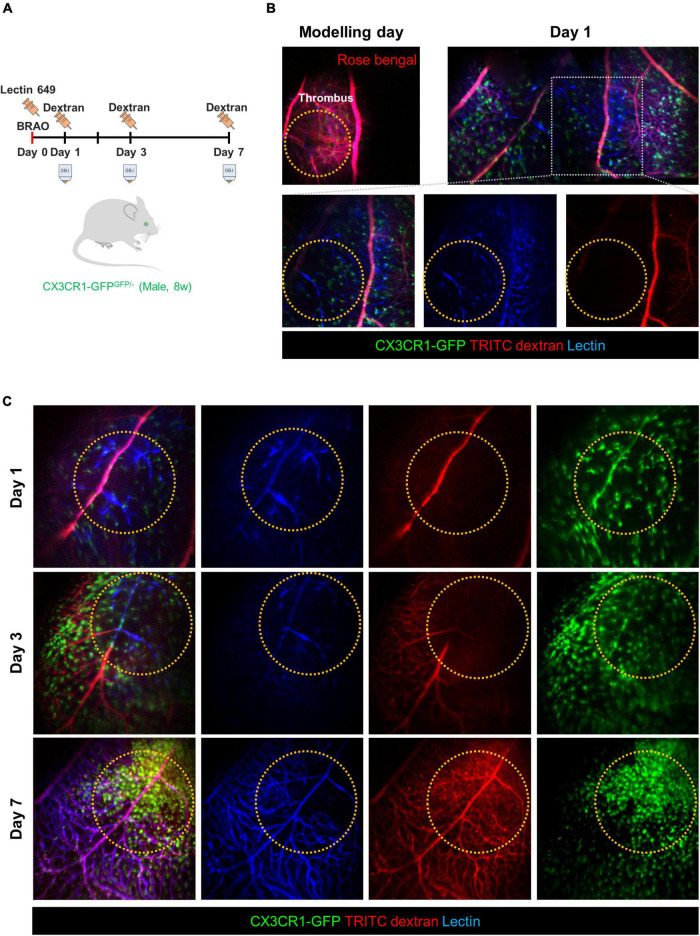
Comparison of the vessel wall and blood flow to confirm the re-perfusion. **(A)** A schematic of experimental plan for vessel staining and dextran blood flow imaging after BRAO modeling. Dextran was injected right before the imaging at the indicated time points of day 1, 3, and 7, respectively. **(B)** Channel split images of the vessel and blood flow test at 1 day after BRAO modeling. **(C)** Serial intravital images of the vessel and blood flow test in same mouse showing that gradual reperfusion for 7 days. BRAO, branch retinal artery occlusion.

The next study was performed to distinguish between the blood vessel walls and actual blood flows in the BRAO models. Figure 3A shows a schematic of the time schedule in this experiment. BRAO was induced by Rose bengal thrombus after intravenous injection of lectin Dylight 649 for vessel wall staining (42). In the following days (days 1, 3, and 7), TRITC-dextran was intravenously injected right before the imaging to visualize actual blood flow. The actual blood flow and retinal vessel walls were easily distinguished by this method, as shown in Figure 3B. This figure shows that obstructed vessels stained by the lectin were still remained, but blood flow could not pass blocked vessels. The state of the blood vessel reperfusion was initiated at day 3 and a total recovery of blood flow at day 7 was confirmed by serial intravital imaging of the actual blood flow, as shown in Figure 3C.

### Activated phenotypes of the CX3CR1-GFP cells after arterial recanalization

**FIGURE 4:**
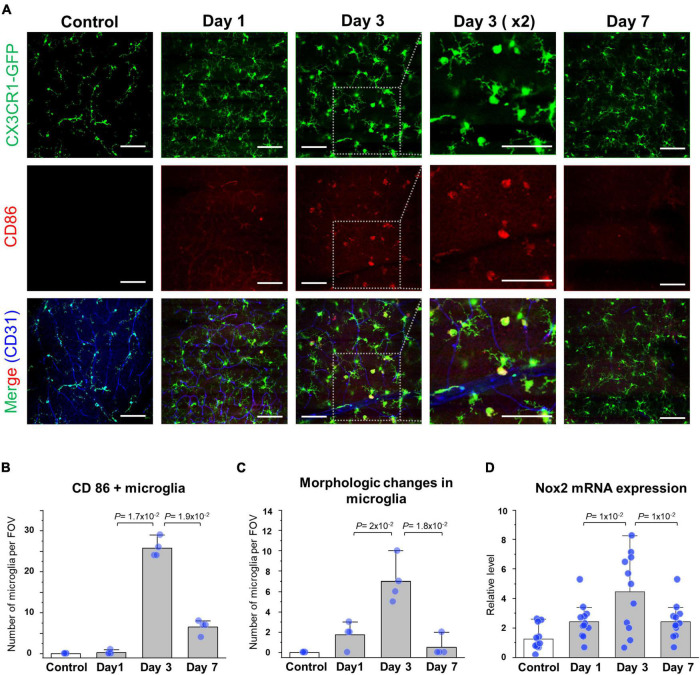
Reperfusion and expression of the microglial activation marker at day 3. **(A)** Serial IHC staining of the CD86 microglial expression marker. *Gray dotted boxes* are magnified CX3CR1, CD86, and CD31 images showing increased CD86 expression around a superficial (Inner limiting membrane to inner plexiform) layer at day 3. Vascular endothelial cells were stained by CD31 antibody and CD86 surface proteins were stained by CD86 antibody. **(B)** Quantification of the number of CD86/CX3CR1 double (+) cell in a field (*n* = 4, retinas per each group). **(C)** Quantification of the number of morphologic changes from the ramified to amoeboid microglia in a field (*n* = 4, retinas per each group). **(D)** mRNA expression level of the Nox2 in BRAO neuro-retina fraction by RT-qPCR (*n* = 12, retinas per each group). *Scale bars*: 100 μm. FOV, field of view.

To analyze cellular-level changes of the CX3CR1-GFP^+^ inflammatory cell including the retinal microglia and monocyte after spontaneous arterial recanalization for 7 days after BRAO modeling, immunohistochemical (IHC) staining and quantitative real-time PCR were performed. Recovery of the flow gradually increased around the BRAO area from day 3, as shown in Figures 2B, 3C. Interestingly, inflammatory cell was most activated at day 3 at the starting time point of recanalization. The morphological changes from ramified to amoeboid form (38, 43, 44) are considered as typical activation markers of the retinal microglia. Figures 4A–C shows that morphologic changes and CD86, one of the microglial activation makers (45), expression changes of the CX3CR1-GFP^+^ cells are also most activated at day 3. The activated inflammatory cells were mostly located around the superficial [Inner limiting membrane (ILM) to inner plexiform layer, IPL] layers. The CX3CR1-GFP^+^ cells located in the deep layer (outer plexiform layer, OPL) was relatively unchanged and their dendrite process were maintained without retraction (Supplementary Figure 1). We then quantified Nox2 RNA expression in the ischemic retina because there were several reports that Nox2 RNA expression is significantly increased in IR injury models, and ganglion cell death can be alleviated by deletion of Nox2 (6, 46). Similarly, the RNA expression of Nox2 peaked at day 3, as shown in Figure 4D. Taken together, the expression of CD86 and Nox2 accompanied by inflammatory cells activation was predominantly observed at day 3 after BRAO modeling, implying the inflammatory cells activation is on the apex at the start time of recanalization.

### The distribution changes of CX3CR1-GFP^+^ cell after recovered perfusion

**FIGURE 5:**
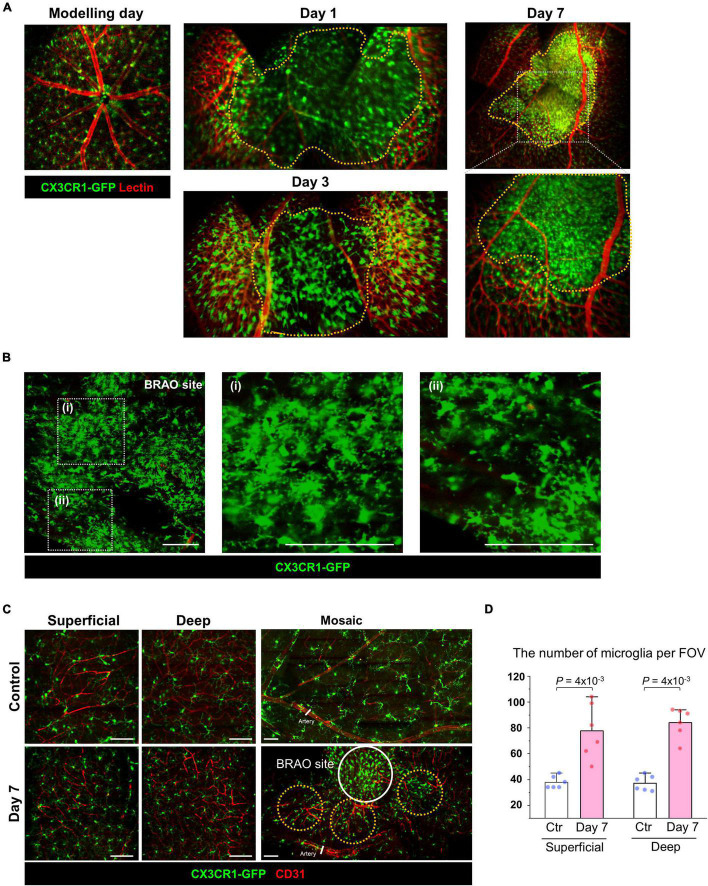
Microglial accumulation after reperfusion at day 7. **(A)** Expanded mosaic image of the intravital CX3CR1-GFP mouse after BRAO modeling. Ischemic lesions were outlined by *orange dotted lines*. **(B)** IHC staining of BRAO site at 7 days after modeling with CX3CR1-GFP mouse. **(C)** IHC staining of the Superficial and Deep (Outer plexiform) layer. IHC samples at peri-BRAO site were collected around *the orange dotted circles*. A core of the BRAO site is indicated by *the white circle*. Vascular endothelial cells were stained by CD31 antibody. **(D)** Retinal microglia numbers in peri-ischemic area in each Superficial and Deep layer. (*n* = 6, respectively) *Scale bars*: 100 μm. FOV, field of view. BRAO, branch retinal artery occlusion.

To analyze the changes of inflammatory cell distribution in BRAO models, an extended mosaic intravital image was captured in CX3CR1-GFP mouse with lectin vessel staining. At day 7, CX3CR1-GFP signals were dramatically increased in the intravital mosaic image with the recovered blood flow in large vessels, as shown in Figures 3C, 5A. Significant inflammatory cell accumulations were observed at the ischemic BRAO site, as shown in Figure 5A. IHC images after BRAO day 7 showed that most of the accumulated CX3CR1-GFP^+^ cells had dendritic process, as shown in Figure 5B. Additionally, it revealed a dramatically increased number of ramified inflammatory cells in both of the superficial and deep layer not only at the BRAO site but also at the peri-ischemic lesion, as shown in Figures 5C,D.

**FIGURE 6:**
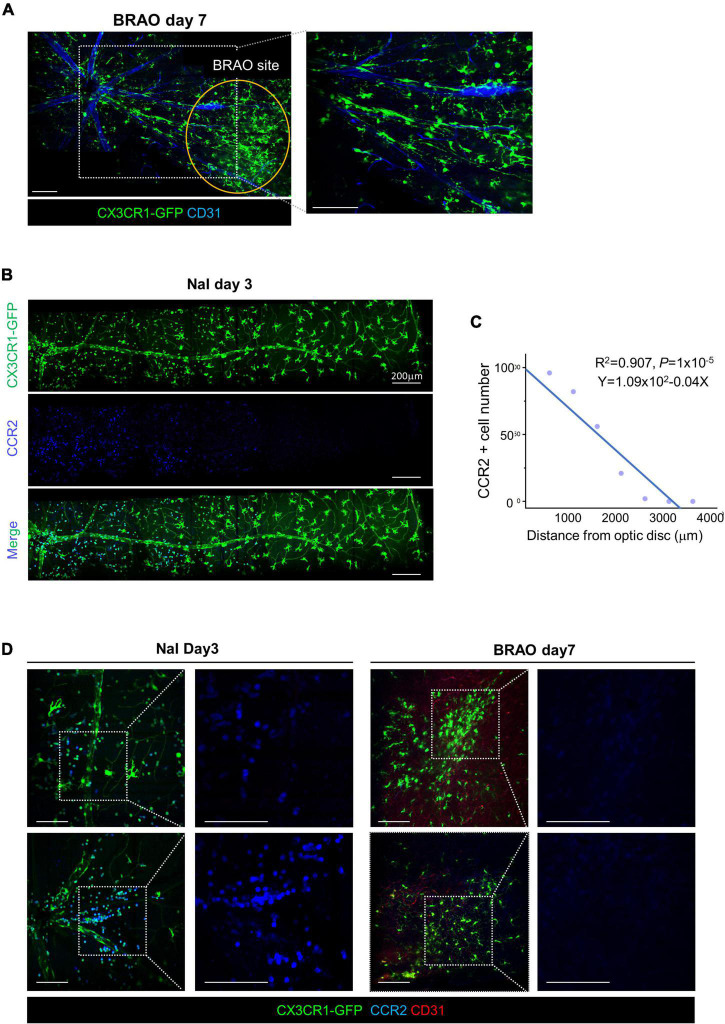
Microglial recruitment from optic disc after BRAO day 7. **(A)** Microglial recruitment from optic disc at day 7 after BRAO, local activation. A core of the BRAO site is indicated by *the orange circle*. Vascular endothelial cells were stained by CD31 antibody. **(B)** CCR2 + Monocytes recruitment from optic disc at day 3 after sodium iodate, systemic activation, *Scale bars*: 200 μm. **(C)** Relationships between the number of monocyte and distance from an optic disc. Monocyte and vascular endothelial cells were stained by CCR2 and CD31 antibody in, respectively. **(D)** IHC staining of CCR2 in each sodium iodate and BRAO showing inflammatory cell composition in CX3CR1 signal. *Scale bars*: 100 μm **(A,D)**. BRAO, branch retinal artery occlusion.

An extend mosaic intravital imaging showed that most of the accumulated CX3CR1-GFP^+^ cells had their dendritic process after BRAO day 7 (Figure 6A). In the case of the systemic activation with BRB break by sodium iodate model, NaI, inducing a significant recruitment of the CCR2^+^ monocyte (Figures 6B,C). In contrast, in the BRAO model, some microglia seems to migrate along a large vein but not a large artery without the recruitment of the CCR2^+^ monocyte (Figure 6D). Additionally, it seemed that some CX3CR1-GFP cell migration was related with Rod-like microglia in axons (47). The observation using Thy1-eYFP-16 mouse, a nerve fiber reporter mouse, evaluated to find out the migration of microglia along the nerve fiber as well as the large vein (Supplementary Figure 2). While the CCR2^+^ monocyte from the systemic circulation is the dominant type of recruited inflammatory cell in the NaI induced systemic activation model, the tissue resident macrophage–“microglia” could occupy some portion in the total population of the observed inflammatory cells near the optic nerve head when local ischemic activation induced by BRAO modeling.

## Discussion

Branch retinal artery occlusion is a focal ischemic disease of the retina. Although it is one of rare diseases, it has been a critical threat for vision. The artery occlusion located around the macular is particularly harmful (48–50). Studies have previously dissected the molecular pathophysiology of retinal arterial disease in hypertensive condition (51), but the insights in to the occlusive etiology of the retinal arterial occlusion is understudied. Moreover, the lack of studies focusing on BRAO has limited our understanding on the pathophysiology of this disease. Establishments of BRAO model and its detailed analysis would provide great benefits to researchers for the following reasons: (1) BRAO site is clearly distinguishable between disease and healthy vessels; therefore, it can be helpful for biological experiments to understand the pathology of the disease vessel. (2) The focal ischemia model was especially useful for studying the immunological reaction of the specific cell dynamics in other organs (52, 53). Therefore, in this study, we established an easily reproducible BRAO mouse model that have more natural pathophysiologic conditions of BRAO patients by using a custom-built retinal confocal microscopy (30, 31). The established method can consistently and reliably induce BRAO with the real-time *in vivo* retinal imaging capability as shown in Figure 1D and Supplementary Movie 1. To note, among total 40 mice used in this study with BRAO modeling with the optimized laser illumination power and duration (27 s, 13.1 mW), there was no failure in the modeling or complications such as vessel rupture. Furthermore, this BRAO modeling method is easily modified to branch retinal venous occlusion (BRVO) modeling by just changing the target vessel.

There is also an increasing demand for emergency reperfusion therapies with the development of relevant medical technology and the intervention technique (54, 55). Recently, intervention therapies are commonly used for rescuing the RAO by using thrombolytics, such as tPA (56, 57). However, even after successful procedures of reperfusion and oxygen supply were achieved, only 17% of the patients could regain functional visual acuity (21). To note, there is a report that some of the patients after tPA reperfusion suffered an IR injury (58). Although tPA reperfusion has a prominent merit to supply oxygen restoration, the rapid reperfusion could cause excessive production of reactive oxygen species inducing harmful effects of retinal function. Nevertheless, until now, there has been a very limited studies about the IR injury of the BRAO because an adequate and clinically meaningful BRAO model has been absent. Establishment of relevant mouse models for BRAO patients may help to study disease pathophysiology, and learning how to regulate inflammatory cell activation and recruitment may provide therapeutic insights for how an IR injury progresses in patients. For the best of our knowledge, this is the first study of the arterial recanalization in BRAO modeling with real-time longitudinal imaging of the retina. Although this study cannot be generalized to the IR injury, it can provide helpful information for further researches in the field.

There are several reports that microglia is involved in the reaction of the IR injury in MI and stroke (24, 25, 59). However, the role of the microglia is still not fully understood and remains obscure with controversies. Furthermore, function of “the retinal microglia” in pathological condition is more ambiguous than CNS microglia. In this study, we serially followed up retina by intravital imaging and IHC histology for observing inflammatory cell activation after arterial recanalization in BRAO during progression for 7 days. Interestingly, a specific microglial activation marker including CD86 and Nox2 (IR injury marker) prominently increased at the moment of reperfusion (Figure 4). And there is also a report that transient ischemia can cause microglia activation in other organs with a similar concept study by using middle cerebral artery occlusion (60). After reperfusion, some dendritic CX3CR1-GFP^+^ cells gradually recruited from the disc through vein and nerve fibers to the BRAO ischemic area (Figure 5 and Supplementary Figure 2). The increase number of inflammatory cell might be partially originated from the external microglia such as the iris, ciliary body and optic nerve microglia, due to the relatively slow repopulation and duplication time of the retinal microglia even in the depletion state (61–63).

Our longitudinal observation suggests that the retinal CX3CR1-GFP^+^ cells may have some effects on the arterial recanalization of the BRAO. Yet, to further improve our understanding of inflammatory response and recovery after arterial recanalization of BRAO, more in-depth analysis in the molecular profiles of the inflammatory cells including retinal microglia is required. Another limitation is that no direct comparison between acute tPA thrombolytic reperfusion and Rose bengal self-reperfusion was performed. Technological development for mouse thrombolytic therapy that can target single BRAO vessels would be needed to tackle this limitation. In addition, this study did not perform a direct comparison between the permanent artery occlusion and transient occlusion by the self-remission of the Rose bengal thrombus. Longer laser illumination to induce a permanent occlusion could cause several complications such as subretinal hemorrhages and vessel ruptures, thereby it would be difficult to analyze the direct effects of the ischemia.

## Conclusion

In this work, we successfully established a reproducible BRAO model by using a custom-built confocal microscopy. This BRAO model can easily control the time point and specifically target a single vessel by optimizing the laser power, projection time and precisely adjusting the AOV. Followed by the successful BRAO modeling, we performed a spontaneous arterial recanalization study of the inflammatory cells in BRAO. Dynamic alteration in the molecular profiles of the immune cell, including microglia, were most prominent at 3 days after BRAO modeling with the reperfusion of thrombus self-resolution. After fully restored reperfusion at day 7, some CX3CR1-GFP^+^ cells were focally recruited and accumulated around the ischemic area. The established method could be a useful tool for investigating the pathophysiology of occlusion retinal diseases.

## Data availability statement

The original contributions presented in this study are included in the article/Supplementary Material, further inquiries can be directed to the corresponding author.

## Ethics statement

The animal study was reviewed and approved by the Institutional Animal Care and Use Committee of Korea Advanced Institute of Science and Technology (KAIST).

## Author contributions

PK has full access to all data in the study and takes responsibility for the integrity of the data and accuracy of the data analysis. JJ, S-HK, SK, and JY conducted the experiments. JJ, EK, JYL, JL, and Y-MK designed the experiments. JJ and PK wrote the manuscript. All authors contributed to the article and approved the submitted version.

## Conflict of interest

The authors declare that the research was conducted in the absence of any commercial or financial relationships that could be construed as a potential conflict of interest.

## Publisher’s note

All claims expressed in this article are solely those of the authors and do not necessarily represent those of their affiliated organizations, or those of the publisher, the editors and the reviewers. Any product that may be evaluated in this article, or claim that may be made by its manufacturer, is not guaranteed or endorsed by the publisher.

## Supplementary material

Supplementary Figure 1 Microglial distribution at each layer after BRAO modeling. **(A)** Representative IHC staining images of the superficial layer and deep layer showing that microglial activation is relatively quiescent at OPL. Vascular endothelial cells were stained by CD31 antibody and CD86 surface proteins were stained by CD86 antibody. **(B)** Number of CD86/CX3CR1 double (+) cell in a field (*n* = 4, retinas per each group). *Scale bars*: 100 μm. S, superficial layer, D, deep layer, FOV, field of view. BRAO, branch retinal artery occlusion.

Supplementary Figure 2 Microglial recruitment through optic nerve and vein in BRAO modeling. *Arrow* indicated the retinal microglia. Vascular endothelial cells were stained by CD31 antibody and microglia were stained by IBA-1 antibody. *Scale bars*: 100 μm. BRAO, branch retinal artery occlusion.

Supplementary Movie 1 Real-time intravital imaging of the branch retinal artery occlusion modeling.
