# Supplementary figures and images for: Establishment of the reproducible branch retinal artery occlusion mouse model and intravital longitudinal imaging of the retinal CX3CR1-GFP+ cells after spontaneous arterial recanalization
Source: Front Med (Lausanne). 2022 Jul 15;9:897800. doi: 10.3389/fmed.2022.897800 (PMC9334526; doi:10.3389/fmed.2022.897800)

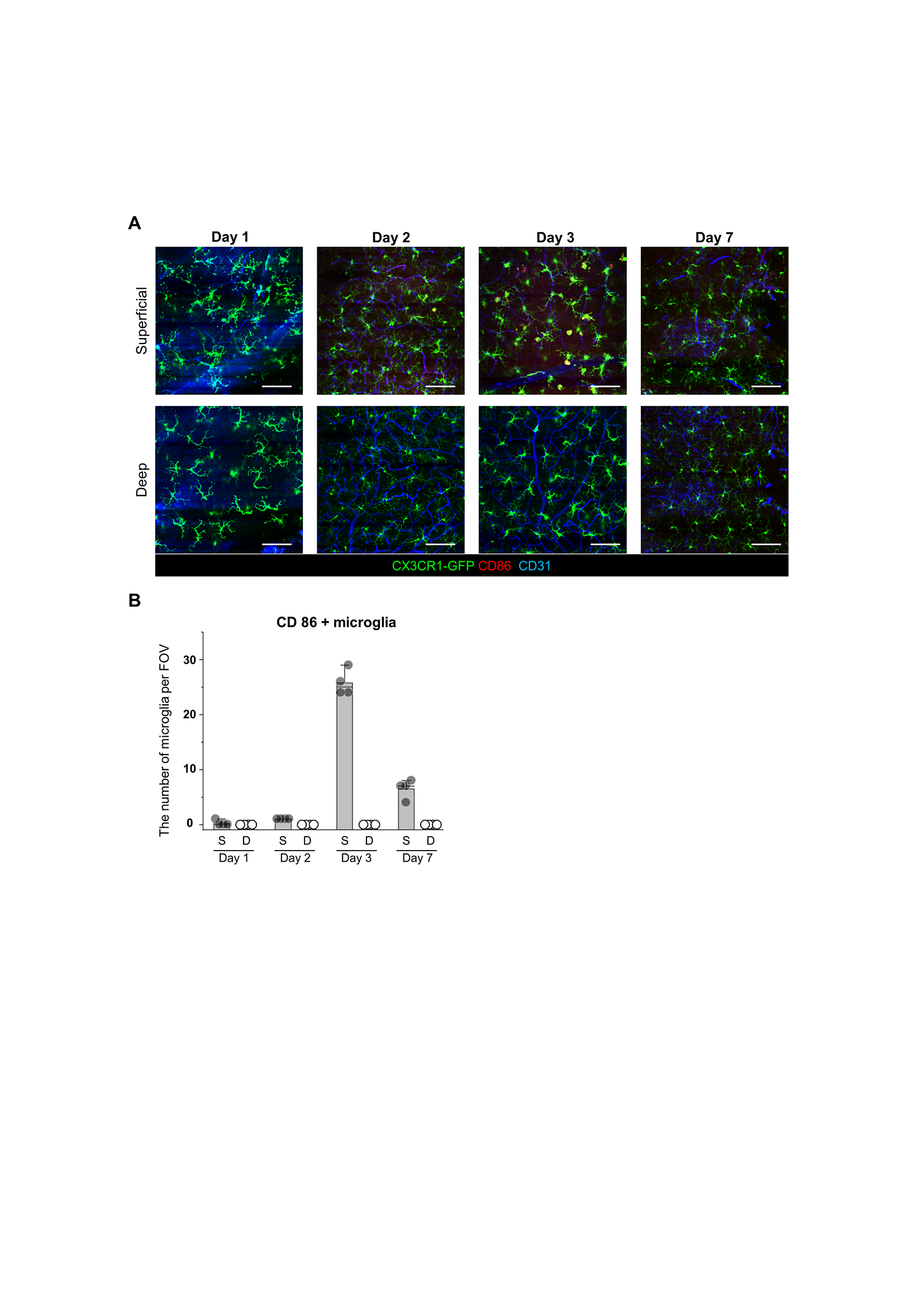

Supplement: Supplementary Figure 1 — Microglial distribution at each layer after BRAO modeling. (A) Representative IHC staining images of the superficial layer and deep layer showing that microglial activation is relatively quiescent at OPL. Vascular endothelial cells were stained by CD31 antibody and CD86 surface proteins were stained by CD86 antibody. (B) Number of CD86/CX3CR1 double (+) cell in a field (n = 4, retinas per each group). Scale bars: 100 μm. S, superficial layer, D, deep layer, FOV, field of view. BRAO, branch retinal artery occlusion. [file Image_1.TIF]

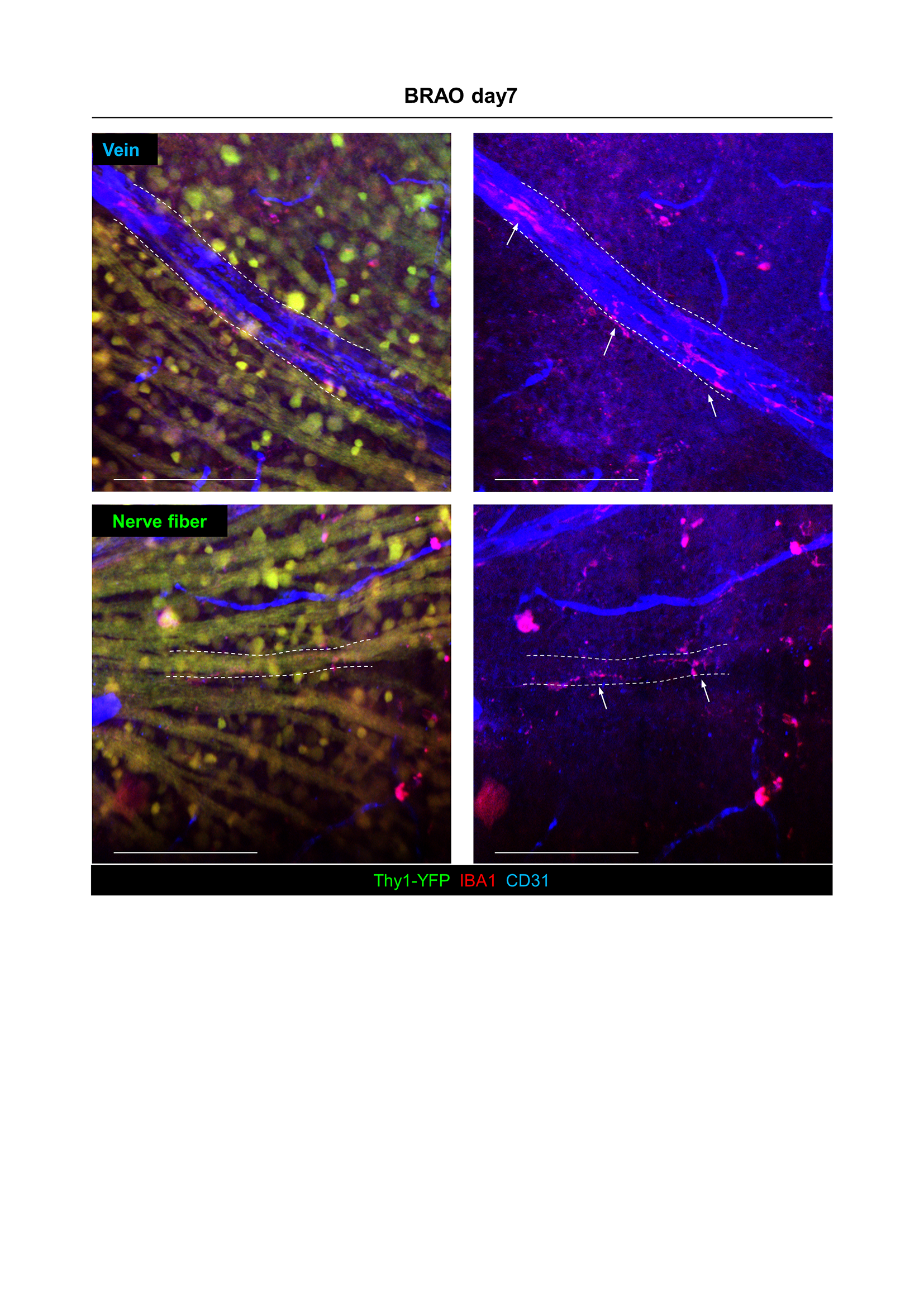

Supplement: Supplementary Figure 2 — Microglial recruitment through optic nerve and vein in BRAO modeling. Arrow indicated the retinal microglia. Vascular endothelial cells were stained by CD31 antibody and microglia were stained by IBA-1 antibody. Scale bars: 100 μm. BRAO, branch retinal artery occlusion. [file Image_2.TIF]
