# Supplementary material for: Establishment of the reproducible branch retinal artery occlusion mouse model and intravital longitudinal imaging of the retinal CX3CR1-GFP+ cells after spontaneous arterial recanalization
Source: Front Med (Lausanne). 2022 Jul 15;9:897800. doi: 10.3389/fmed.2022.897800 (PMC9334526; doi:10.3389/fmed.2022.897800)
Supplement: Supplementary file 4 [file Table_1.DOCX]

# Supplementary table1. Quantitative RT-PCR primers for mRNA of the mouse retina

| Gene name | Purpose |  | GC ratio (%) | Annealing temperature |
| --- | --- | --- | --- | --- |
| Nox2 | Forward primer (5’to3’) | TCAAGACCATTGCAAGTGAACAC | 43 | 60 |
|  | Reverse Primer (3’to5’) | TCAGGGCCACACAGGAAAA | 53 | 60 |
| GAPDH | Forward primer (5’to3’) | AACTTTGTGAAGCTCATTTCCTGGTAT | 37 | 60 |
|  | Reverse Primer (3’to5’) | CCTTGCTGGGCTGGGTGGT | 68 | 60 |
